# Supplementary material for: Copper acquisition in Bacillus subtilis involves Cu(II) exchange between YcnI and YcnJ
Source: bioRxiv. 2025 May 27:2025.05.23.655588. Preprint. [Version 1] doi: 10.1101/2025.05.23.655588 (PMC12154828; doi:10.1101/2025.05.23.655588)
Supplement: Supplement 1 [file media-1.pdf]

Supporting Information for:

Copper acquisition in *Bacillus subtilis* involves Cu(II) exchange between YcnI and YcnJ

Yuri Rafael de Oliveira Silva[a], Grayson Barnes[b], Dia Zheng[a], Daniel Zhitnitsky[e], Samuel Geathers[a],

Stephen C. Peters[c], Veronika A. Szalai[d], John D. Helmann[b], Oriana S. Fisher[a,e*]

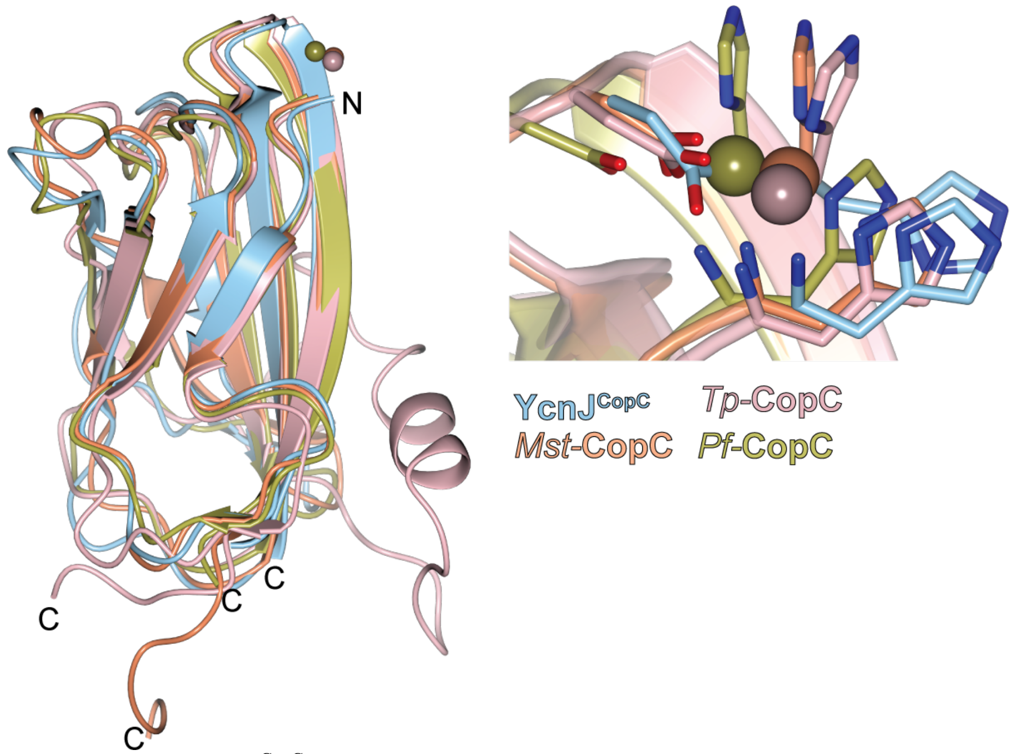

Fig. S1. Superposition of YcnJ^{CopC} (blue) with other experimentally determined structures of Cu(II)-bound CopC proteins (PDB IDs 5ICU – from *Methylosinus trichosproium OB3b* [13], 6NFQ – from *Pseudomonas fluorescens* [17], 8YTR – from *Thioalkalivibrio paradoxus Arh1*) and their Cu(II)-binding sites as an inset.

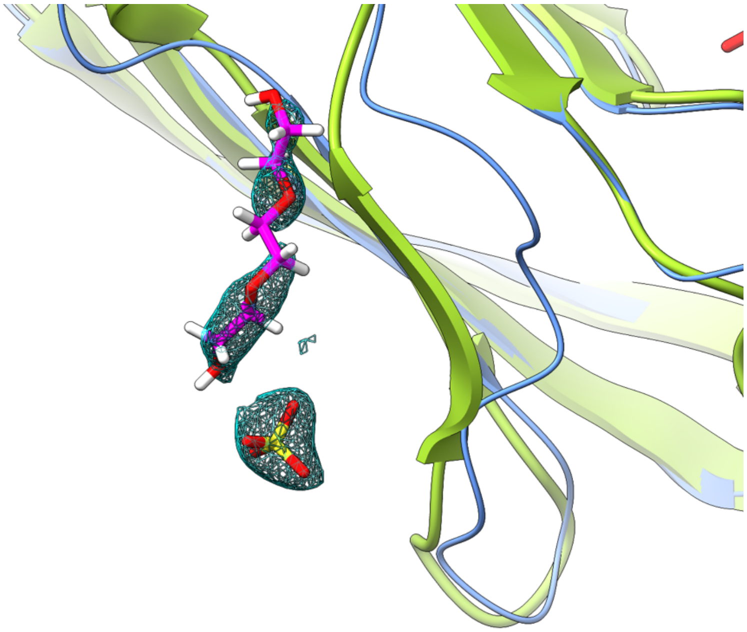

Figure S2. Strand displacement is a crystallographic artifact. A β-strand present in other CopC proteins, such as that from *M. trichosporium OB3b* (PDB ID 5ICU, yellow green) is displaced by the presence of triethylene glycol and a sulfate ion (magenta and yellow, respectively) in the structure of $YcnJ^{CopC}$ (cornflower blue). $2F_o$-F_c maps of triethylene glycol and SO_4 are shown (contour level: 0.30).

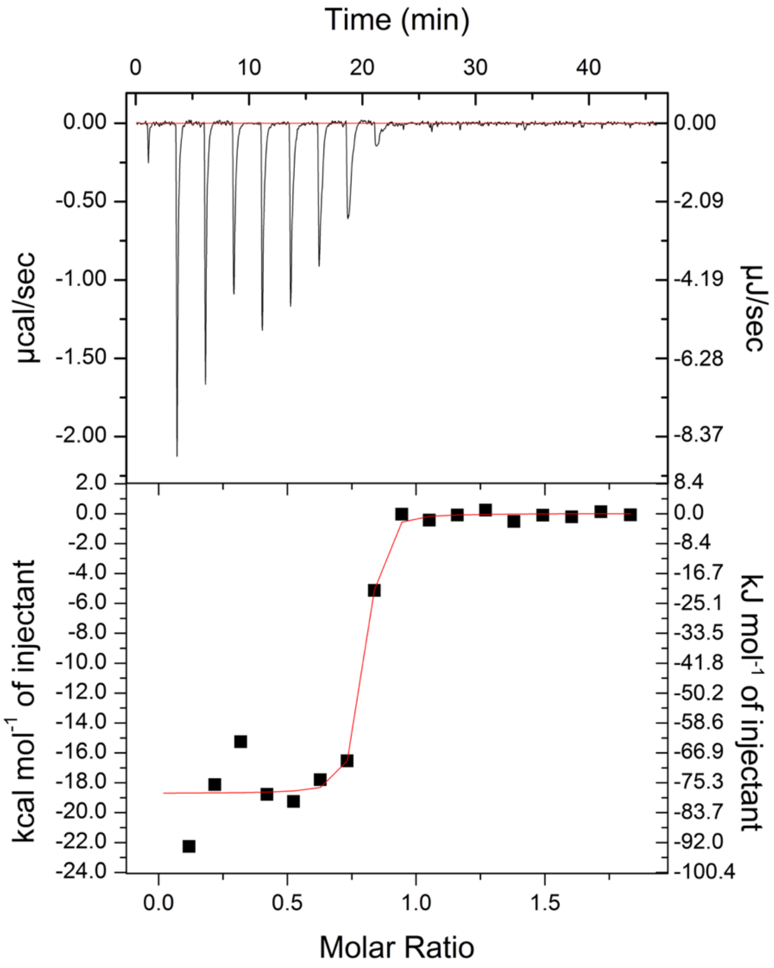

Figure S3. Replicate experiment of isothermal titration calorimetry of Cu(II) into YcnJ^{CopC}. Measurements were performed in the presence of 30 mmol/L glycine as a weak competitor. Conditional $K_D = 1.32 \times 10^{-16}$ mol/L; $n_{ITC} = 0.752 \pm 0.0121$; $\Delta H = -78\ 212 \pm 2\ 495$ J/mol; $\Delta S = -120$ J/mol/K. Reported error represents error on the curve fit.

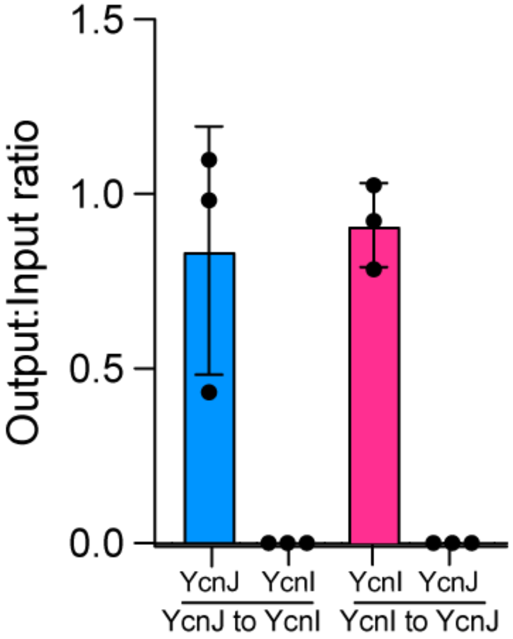

Fig. S4. Cu cannot passively transfer between YcnJ and YcnI. Average ratios of Cu(II) concentrations before and after dialysis of Cu(II)-YcnJ^{CopC} with as-purified YcnI^{DUF1775} (blue) and Cu(II)-YcnI^{DUF1775} with as-purified YcnJ^{CopC} (magenta). For as-purified proteins, only the results after dialysis are shown. The experiment was repeated 3 times. Individual results are shown as black circle and error bars represent calculated SD.

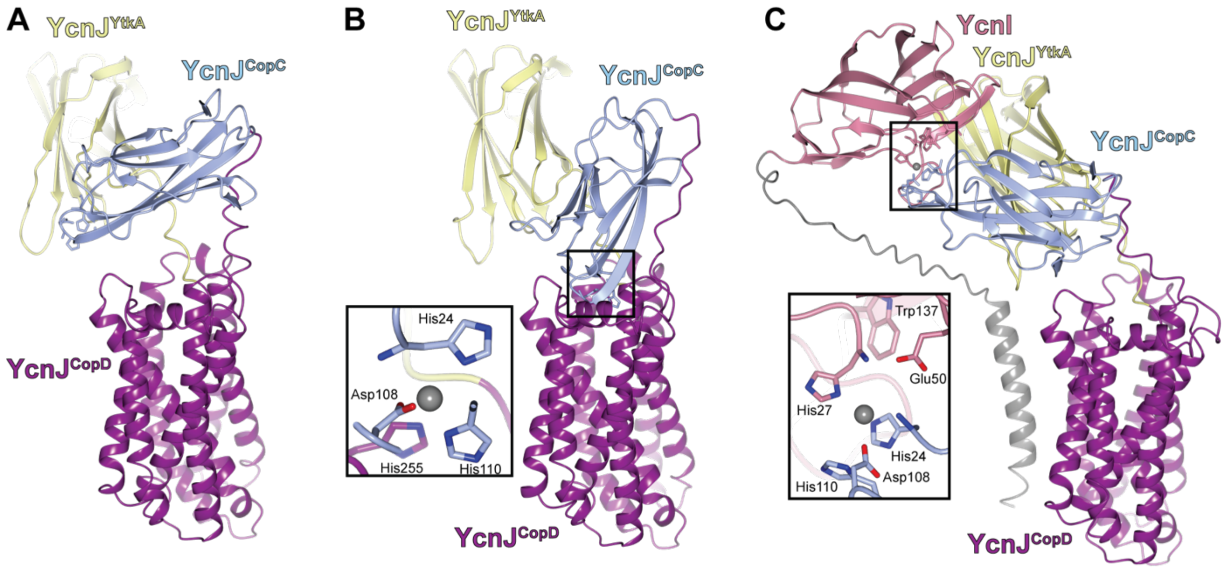

Figure S5. Structural predictions of full-length YcnJ. AlphaFold3 predictions for (a) full-length apo YcnJ, (b) full-length YcnJ with 1 Cu(II) ion, and (c) full-length YcnJ in complex with full-length YcnI and 1 Cu(II) ion. Proteins are colored by domain, and insets represent predicted locations for the Cu(II) ion.

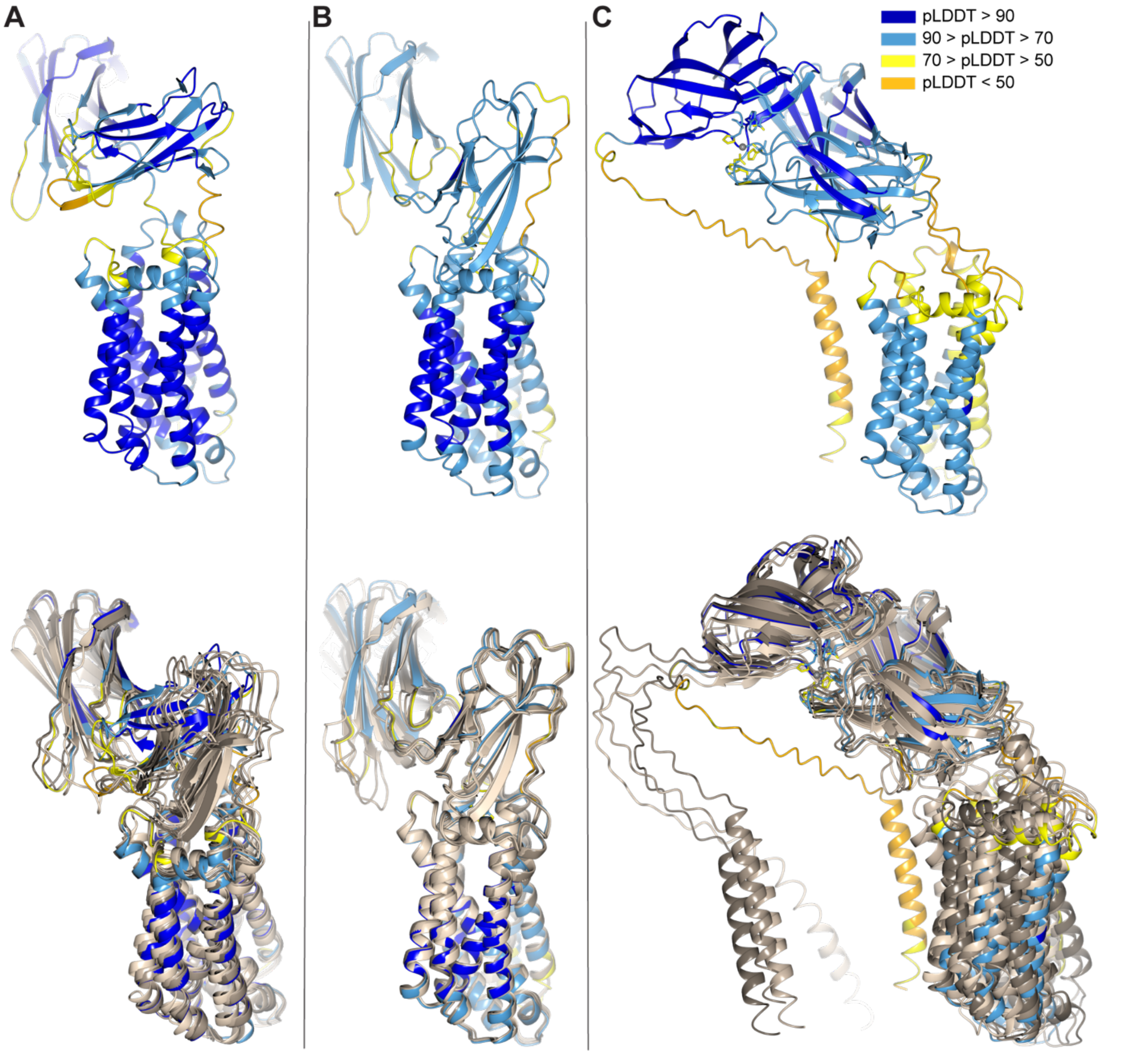

Figure S6. AlphaFold3 predictions. The same AlphaFold3 models from Fig. S4 colored by pLDDT values (top) and superposition of the five best models output (bottom). (A) full-length apo YcnJ, (B) full-length YcnJ with 1 Cu(II) ion, and (C) full-length YcnJ in complex with full-length YcnI and 1 Cu(II) ion.

Table S1. Data collection and refinement statistics

Crystal	$YcnJ^{CopC}$
PDB accession code	9C14
Data collection	
Wavelength (Å)	1.378
Space group	$P4_32_12$
Cell dimensions	
a, b, c (Å)	36.0, 36.0, 165.8
α, β, γ (°)	90.0, 90.0, 90.0
Resolution (Å)*	41.44 to 1.60 (1.63 to 1.60)
R_{meas}*	0.876 (40.355)
$CC_{½}$*	0.997 (0.516)
I / σI*	9.6 (1.00)
R_{merge}*	0.858 (-)
R_{pim}*	0.177 (8.103)
Completeness (%)*	100.0 (100.0)
Redundancy*	24.1 (24.6)
Refinement	
Resolution (Å)*	41.44 to 1.60 (1.65 to 1.60)
No. of reflections*	15 313 (1 209)
R_{work} / R_{free} (%)*	21.1 / 23.3 (47.5 / 48.6)
Residue range built	24 to 120
No. of atoms	
Protein	789
Ligand/ion	10 SO_4, 24 PGE
Water	62
Model Quality	
B-factors ($Å^2$)	
Overall	24.00
Protein	35.55
Ligand/ion	46, 48, 30
Water	
RMSD, bond lengths (Å)	
RMSD, bond angles (°)	
Ramachandran favored/allowed/outliers (%)	95 / 2 / 0

*Parentheses indicate highest resolution shell.
Note that 1 nm = 10 Å

Table S2. Strains used in this study

Strain	Genotype	Construction	Reference
B. subtilis			
CU1065	*WT*	Lab strain	Lab stock
HB30921	*ΔycnJ::erm*	BGSC gDNA-->CU1065	This work
HB30922	*ΔycnI::erm*	BGSC gDNA-->CU1065	This work
HB30927	*ΔycnJ*	pDR244-->HB30921	This work
HB30930	*ΔycnI*	pDR244-->HB30922	This work
HB30956	*ycnI (His27Ala)*	CRISPR (pAJS23+repair template)--> HB30922	This work
HB30958	*ycnI (Glu50Ala)*	CRISPR (pAJS23+repair template)--> HB30922	This work
HB30959	*ycnI (Trp137Phe)*	CRISPR (pAJS23+repair template)--> HB30922	This work
HB30927	*ycnI (TruncAsp170)*	CRISPR (pAJS23+repair template)--> HB30922	This work
HB30960	*ycnJ (His24Ala)*	CRISPR (pAJS23+repair template)--> HB30921	This work
HB30977	*ycnJ (His110Ala)*	CRISPR (pAJS23+repair template)--> HB30921	This work

Table S3. Conditional Dissociation Constants (K_D) of binding of CopC proteins to Cu(II).

Protein	K_D (M)	n	Reference
$YcnJ^{CopC}$ (1)	1.43×10^{-16}	0.682	This study
$YcnJ^{CopC}$ (2)	1.32×10^{-16}	0.752	This study
$YcnI^{WT}$	3.51×10^{-15}	1.07	de Oliveira Silva et al. (23)
$YcnI^{W137F}$	2.02×10^{-14}	0.35	de Oliveira Silva et al. (23)
YobA	3×10^{-9}	1.09	Hadley et al. (15)
*Pf*CopC*	10^{-16}	-	Wijekoon et al (20)
*Ps*CopC*	10^{-14}	-	Wijekoon et al. (20)<br>Zhang et al. (26)

*Constants measured via ligand competition using fluorescent probes.
